# Development of superluminal pulse propagation in a serial array of high-Q ring resonators

**DOI:** 10.1038/s41598-019-50482-9

**Published:** 2019-10-03

**Authors:** Yuma Morita, Makoto Tomita

**Affiliations:** Department of Physics, Faculty of Science, Shizuoka University, 836, Ohya, Suruga-ku, Shizuoka 422-8529 Japan

**Keywords:** Optical physics, Optical physics

## Abstract

We experimentally examined the development of superluminal pulse propagation through a serial array of high-Q ring resonators that provides a dynamic recurrent loop. As the propagation distance, i.e., the number of ring resonators that the pulses passed through increased, the pulse advancement increased linearly, largely maintaining its Gaussian shape. The sharp edge encoded at the front of the pulse was, however, neither advanced nor delayed, in good accordance with the idea that information propagates at the speed of light. We also carried out a numerical simulation on the superluminal to subluminal transition of the pulse velocity, which appeared after the pulse had propagated a long distance. The time delays, which we calculated using the saddle point method and based on the net delay, were in good agreement with our results, even when predictions based on the traditional group delay failed completely. This demonstrates the superluminal to subluminal transition of the propagation velocity.

## Introduction

Coherent wave propagation through resonators sometimes resembles that in atoms. Normal and anomalous dispersions in resonators, similar to those in atomic absorption lines, create slow and fast light, respectively^1–4^. Coupled-resonator-induced transparency^5,6^ may be described as analogous to electromagnetically induced transparency in a three-level atomic system^7–9^ and realizes slow light.

In the context of analogues of pulse propagation in atomic and resonator systems, a single-stage resonator corresponds to a single atom, because electromagnetic waves interact only once with the resonator. To emulate the propagation effects over a certain distance in resonator systems, we must prepare a serial array of resonators. Side-coupled integrated sequences of spaced optical resonators (SCISSORs)^10^ and coupled resonator optical waveguides^11,12^ are typical examples of coupled resonators. SCISSORs consist of a chain of resonators that are coupled via a side-coupled waveguide. These structures slow down the propagation velocity and have applications in fundamental physics^13^ and telecommunication systems^4,10–12,14^. The major problem in realizing chains of resonators, however, lies in the fabrication of many identical, low-loss resonators^15^.

An alternative approach to investigating the physics underlying pulse propagation through serial arrays of resonators may be to use a recently developed method based on a dynamic recurrent system^16,17^. Using this method, we can realize an equivalent setup of serial-array resonators consisting of perfectly identical resonance frequencies with ultra-high Q (10^9^) values and losses.

Here, we investigated the development of the propagation of a Gaussian-shaped temporal pulse through a serial array of high-Q ring resonators that provides a dynamic recurrent system^16^. Specifically, our interest lies in the analogy between fast light in atomic and resonator systems. Fast light has been observed in a wide variety of systems^1–3,18–27^. Resonators can also yield steep dispersions to achieve fast light. Here we examined how the negative group delay accumulates as the number of ring resonators *N* that the pulses pass through increases. It has been demonstrated experimentally that as *N* increases, the advancement of the pulse peak increases linearly. The sharp edge encoded at the front of the pulse, however, is neither advanced nor delayed; this is in good accordance with the idea that the non-analytical point behaves as information^21–27^. We also discuss the superluminal to subluminal transition in the pulse velocity^28–30^ based on the results of our numerical simulations. We developed an analogous saddle point method^28,31^, as well as the concept of net and reshaping delays^32,33^. This approach, originally discussed in the context of continuous media (atomic system), is also relevant to a serial array of high-Q ring resonators. The time delays calculated using these methods were in good agreement with our results, even when the concept of traditional group velocity failed completely.

## Experiments

### Peak and front velocities

**Figure 1:**
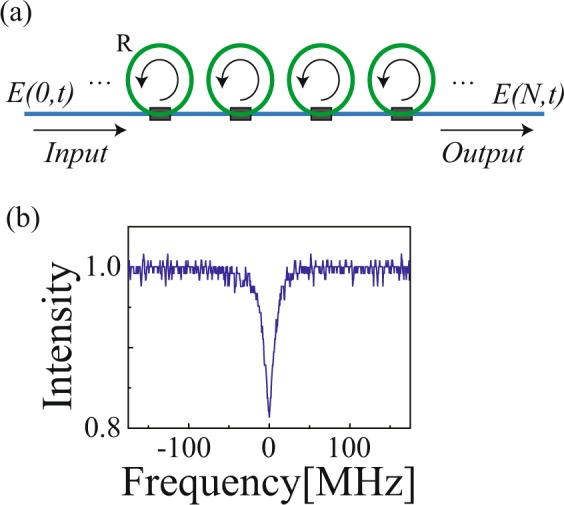
(**a**) Conceptual illustration of a side-coupled multi-stage serial array of ring resonators. *R* is the ring resonator. (**b**) Transmission spectra as a function of the detuning frequency in a ring resonator (single-stage).

The concept of the serial array of ring resonators is illustrated in Fig. 1(a), where high-Q ring resonators are coupled through a side waveguide. The input–output characteristics of the single-stage ring resonator have been well studied^3^. The output light intensity $T(\omega )$ shows a dip as a function of *N*. The response function is given by

1 $$\begin{array}{rcl}{\rm{Res}}(\omega ,x,y) & = & |T(\omega )|{e}^{i\theta (\omega )}\\  & = & {(1-\gamma )}^{\frac{1}{2}}\frac{y-x\exp [i\varphi (\omega )]}{1-xy\exp [i\varphi (\omega )]}\end{array},$$

where $\gamma$ is the excess loss at the coupler, *ϕ*(*ω*) is the phase shift given by $\phi (\omega )=\frac{{L}_{R}}{c}\omega$, ${L}_{R}$ is the optical length of the ring resonator, and *c* is the light velocity. The dispersion relationship depends on the loss parameter *x*, and the coupling parameter *y*. For the under-coupling condition $(x < y)$, the transmission phase $\theta (\omega )$ exhibits anomalous and normal dispersion at the center and wing regions of resonances, respectively, similar to the absorption lines in atomic systems. In the anomalous dispersion region, the group delay is negative, ${\tau }_{g}=\partial \theta /\partial \omega  < 0$, and superluminal pulse propagation appears. In contrast, for the over-coupling condition $(x > y)$, the transmission phase monotonically shows normal dispersion. In the current study, a 93:7 coupler was used to achieve under-coupling. The length of the ring *L*_*R*_ was 1.0 m. The resonance width, $\Delta {\nu }_{R}$ was 14.4 MHz. Figure 1(b) shows the transmission spectra as a function of the detuning frequency $\nu =\omega /2\pi$ in the single-stage ring resonator.

**Figure 2:**
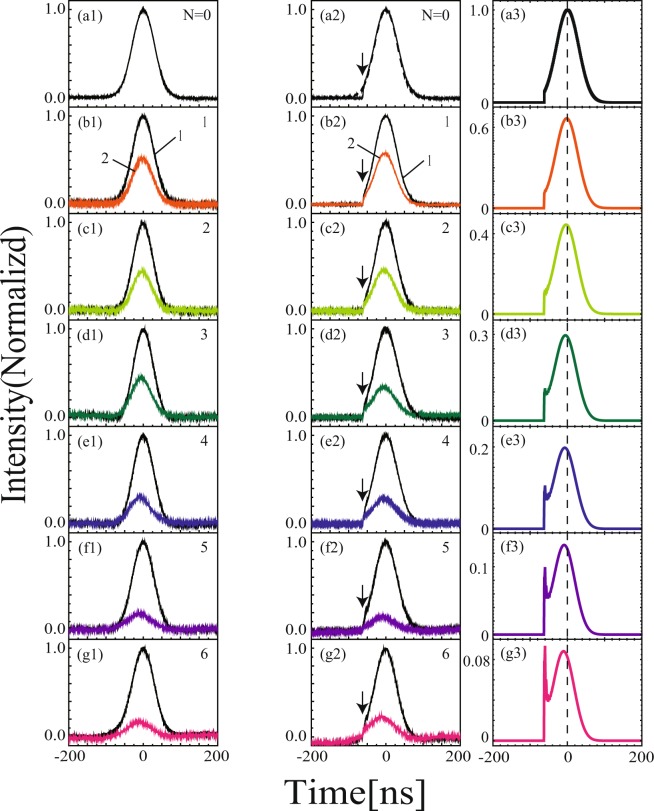
Development of the temporal profiles of pulses transmitted through the serial array of ring resonators for different values of *N*. The left column shows experimental results for the smooth Gaussian-shaped pulse. The middle and right columns are experimental observations and numerical calculations, respectively, for the Gaussian-shaped pulse, on which the sharp front edge was encoded on the leading side of the pulses. (**a**) *N* = 0 (input pulse), (**b**) 1, (**c**) 2, (**d**) 3, (**e**) 4, (**f**) 5, and (**g**) 6. The solid black (line 1 in b1) and colored lines (line 2) are input (off-resonance) and transmitted (on-resonance) pulses, respectively. The intensity of the input pulse was normalized by 1 and the transmitted pulse intensity was scaled with respect to the input pulse. The downward arrows indicate the positions of the edges.

**Figure 3:**
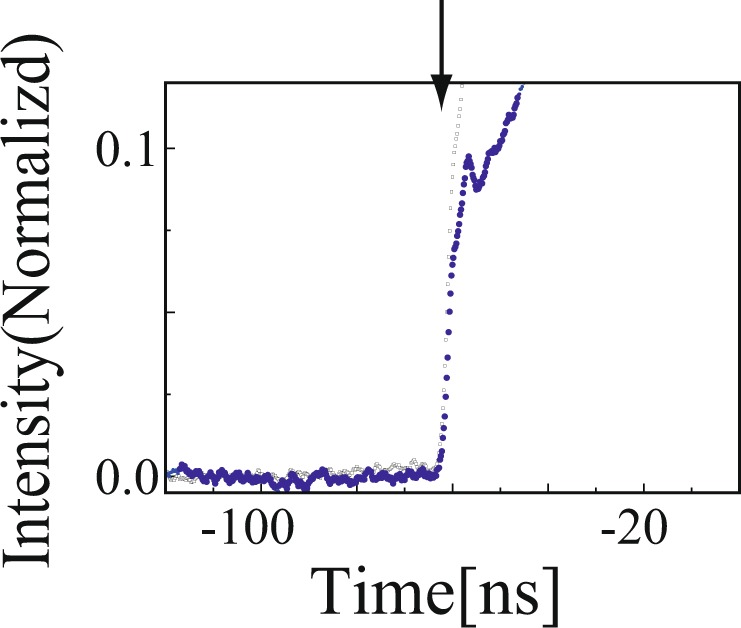
(**a**) Expanded plot of Fig. 2(e2) (i.e., *N* = 4) around the edge region. The dotted black and blue lines are input and transmitted pulses, respectively. The downward arrows indicate the positions of the edges.

**Figure 4:**
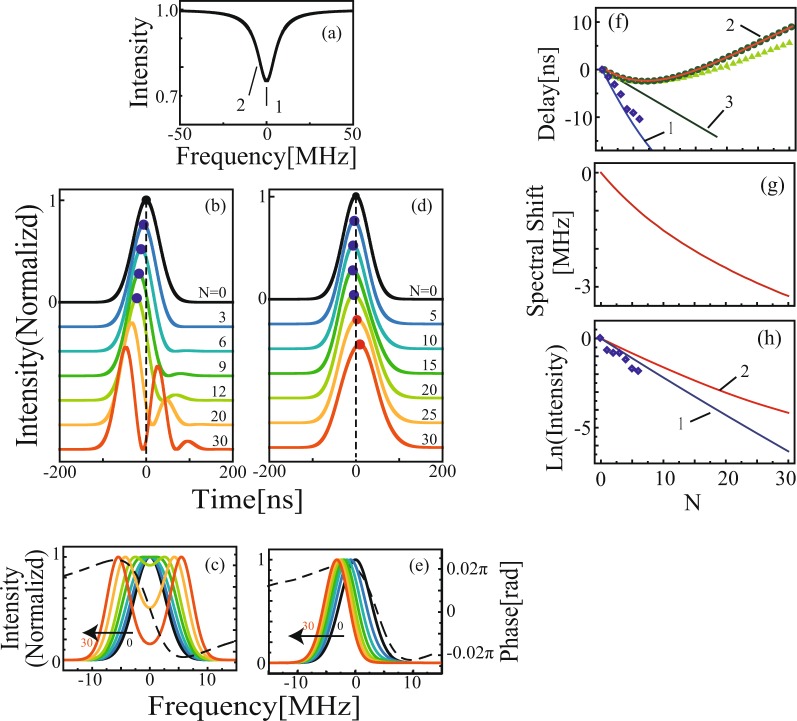
Calculated curves of the pulse propagation through a serial array of high-Q ring resonators. (**a**) Transmission spectra as a function of the detuning frequency in the single-stage ring resonator. Arrows 1 and 2 indicate the frequencies at which the pulse propagation was analyzed. (**b**,**d**) Transmitted temporal profiles of the smooth Gaussian-shaped pulses for different *N*. (**b**) The wavelength of the laser was adjusted to the resonance of the ring resonator [arrow 1 in (**a**)]. (**d**) The frequency was slightly detuned from resonance [arrow 2 in (**a**)]. (**c**,**e**) Transmitted spectral profiles for the smooth Gaussian-shaped pulses for different *N* under the conditions shown in (**b**) and (**d**), respectively. (**f**) Solid blue (line 1) and red (line 2) lines (line 2 is overlapped with the green open circles) represent the delay (advancement) of the center of mass of the pulses as a function of *N* under the conditions shown in (**b**) and (**d**), respectively. The green solid triangles and open circles represent the delay calculated using the saddle point method ${\tau }_{s}$ and are based on the net delay ${\tau }_{net}$, respectively. The solid green line (line 3) shows the expected time delay if the pulse had propagated with the conventional group velocity. The open blue diamonds are the experimental results shown in Fig. 2. (**g**) The solid red line represents the spectral peak as a function of *N* under the conditions shown in (**e**). (**h**) The solid blue (line 1) and red (line 2) lines are the pulse intensity as a function of *N* under the conditions shown in (**b**) and (**d**), respectively. The open blue diamonds indicate the experimental results shown in Fig. 2.

We examined how the negative group delay accumulates as a function of the number of ring resonators *N* using the dynamic recurrent system described in Section Method. Figures 2 and 3 show the experimental results of the pulse profiles transmitted through the serial array of ring resonators. The temporal duration of the Gaussian pulses was ${t}_{p}$ = 60 ns. The colored lines in the left column of Fig. 2 show the transmitted pulse after passage through the ring resonator for different *N*. The wavelength of the laser was adjusted to the resonance of the ring resonator. The time of origin was taken at the pulse peak position observed in the off-resonance condition. Figure 2(a1) shows *N* = 0, i.e., the input Gaussian pulse. Figure 2(b1) shows the transmitted pulse profile for *N* = 1. As the ring resonator was prepared in the under-coupling condition, the pulse peak was advanced by ${\tau }_{g}$ = 1.56 ns. The open blue diamonds in Fig. 4(f) represent the temporal advancement of the peak as a function of *N*. As the number of ring resonators increased, the advancement of the pulse peak increased linearly by a constant value of 1.63 ns/stage. The open blue diamonds in Fig. 4(h) show the normalized pulse intensity as a function of *N*. The pulse intensity decayed exponentially as a function of *N*, ${I}_{N}={I}_{0}\exp [\,-\,N/{N}_{\alpha }]$, where ${N}_{\alpha }$ is the number of stages in which the incident intensity decays to 1/*e* of the initial value, with a constant of ${{N}_{\alpha }}^{-1}$ = 0.33/stage, obeying the conventional Beer–Lambert law in absorbing media.

The superluminal pulse velocity seemingly contradicts the causality of special relativity. However, we can predict the arrival of the pulse peak via an expansion of the leading part of the pulse; hence, the pulse peak contains no new information. It is now understood that the true information is carried by the non-analytical points, such as the pulse front edge^21–27^. Since the pioneering work conducted in this area^18^, the propagation of non-analytical points has been investigated in different systems. To date, the non-analytical point in resonators has only been studied in a single-stage resonator. Here, we examined how the non-analytical point develops with the number of ring resonators *N*. For this purpose, we prepared a Gaussian-shaped intensity profile and encoded a non-analytical point *t*_*NA*_ within the pulse envelope as

$${\tilde{E}}_{in}(t)=\Theta (t)\exp [-{(\frac{t}{{t}_{a}})}^{2}]$$

with

2 $$\Theta (t)=\{\begin{array}{cc}0 & t < {t}_{NA}\\ 1 & t\ge {t}_{NA}\end{array}\,{\rm{o}}{\rm{r}}\,\{\begin{array}{cc}1 & t < {t}_{NA}\\ 0 & t\ge {t}_{NA}\end{array},$$

where ${\tilde{E}}_{in}(t)$, the slowly varying envelope of the pulse $\Theta (t)$, is a Heaviside function.

The colored lines in the middle column of Fig. 2 represent the transmitted pulse profile for a Gaussian-shaped pulse with a sharp edge as a function of the number of passages *N*. The encoding time ${t}_{NA}$ was −62 ns. The pulse peak was still advanced, as was the smooth Gaussian pulse. The advancements of the pulse peak were the same as the smooth Gaussian-shaped pulse for different *N*. This occurred because the encoding time of the leading edge was sufficiently earlier than the group delay ${\tau }_{g}(N)$, i.e. $|{t}_{NA}| >  > |{\tau }_{g}(N)|$; hence, the edge did not affect the peak significantly^34^. Figure 3 shows the expanded plot of Fig. 2(e2), i.e., *N* = 4 around the edge region. The sharp edge was neither advanced nor delayed. The present experiment thus confirms the accumulation of the negative group delay; however, non-analytical points did not appear to be affected by the increase in the number of ring resonators *N*. The rise and fall times of the edges were 2.5 ns; hence, this point contained Fourier components up to 400 MHz. The bandwidth of the ring resonator was $\Delta {\nu }_{R}$ ~ 14.4 MHz. Therefore, step structures with Fourier components greater than that at 14.4 MHz could act as non-analytical points for the present ring resonator.

The right column in Fig. 2 shows the calculation of the transmitted pulse profiles, in which the spectral width of the non-analytical point was assumed to be infinite. In the calculations, a large transient spike appears at non-analytical point ${t}_{NA}$; this corresponds to the resonance precursor^35,36^. In our experiments, this spike was weak. We can attribute this difference to the finite rise time of 2.5 ns in the non-analytical points in our experiments. For comparison, we also calculated the transmitted pulse profile, in which a finite rise time was taken into account. In this calculation, the transient spike appeared small.

### Traditional group velocity

We developed the concept of traditional group velocity ${v}_{g}$ discussed in Lorentz media to describe the pulse propagation in a serial array of ring resonators. The slowly varying envelope of the pulse that passed through *N* ring resonators is denoted as $\tilde{E}(N,t)$; hence, $\tilde{E}(0,t)$ represents the input pulse ${\tilde{E}}_{in}(t)$. In the present system, we may relate the electric field $E(N,\omega )$ to $E(N-1,\omega )$ as

3 $$E(N,\omega )=E(N-1,\omega ){\rm{Res}}(\omega ),$$

where $E(N,\omega )$ is the Fourier transform of $E(N,t)=\tilde{E}(N,t){e}^{-i{\omega }_{0}t}$ and ${\omega }_{0}$ is the carrier frequency of the input pulse. The temporal pulse profile after passing the *N*-stage ring resonator is given by

4 $$\begin{array}{ccc}\mathop{E}\limits^{ \sim }(N,t) & = & \frac{1}{\sqrt{2\pi }}\int E(N,\omega ){e}^{-i(\omega -{\omega }_{0})t}d\omega \\  & = & \frac{1}{\sqrt{2\pi }}\int E(0,\omega ){\{{\rm{R}}{\rm{e}}{\rm{s}}(\omega )\}}^{N}{e}^{-i(\omega -{\omega }_{0})t}d\omega \\  & = & \frac{1}{\sqrt{2\pi }}\int \int E(0,\omega )\\  &  & exp[\,-\,i\omega (t-N{n}_{Ring}(\omega ))]d\omega \end{array}.$$

Here, we introduce an artificial refractive index for the serial array of ring resonators:

5 $${n}_{Ring}(\omega )\equiv \frac{\log \,[{\rm{Res}}(\omega )]}{i\omega }.$$

Under the condition $\delta {\nu }_{p} <  < \Delta {\nu }_{R}$, where $\delta {\nu }_{p}$ is the bandwidth of the incident pulse, the refractive index may be expanded around the incident frequency ${\omega }_{0}$ as

6 $${n}_{Ring}(\omega )\omega =\eta ({\omega }_{0}){\omega }_{0}+{\frac{\partial \eta (\omega )\omega }{\partial \omega }|}_{{\omega }_{0}}(\omega -{\omega }_{0})+\cdot \cdot +i\kappa ({\omega }_{0}){\omega }_{0},$$

where $\eta (\omega )$ and $\kappa (\omega )$ are the real and imaginary parts, respectively, of the artificial refractive index defined by Eq. (5). When $\delta {\nu }_{p} <  < \Delta {\nu }_{R}$, the expansion converges very rapidly. The pulse profile after transmitting through the serial array of ring resonators is given by

7 $$\begin{array}{rcl}\tilde{E}(N,t) & = & exp[\,-\,\kappa ({\omega }_{0}){\omega }_{0}N]\\  &  & exp[i\eta ({\omega }_{0}){\omega }_{0}]\tilde{E}(0,t-N{\frac{\partial \eta (\omega )\omega }{\partial \omega }|}_{{\omega }_{0}})\end{array}.$$

Equation (7) indicates that the pulse propagates through the serial array of ring resonators without significant pulse deformation. In this case, the group velocity defined by Eq. (6) provides a good picture of the superluminal pulse propagation, as long as *N* is sufficiently small. From Eq. (7), the pulse peak is delayed by ${\tau }_{N}=N{\frac{\partial \eta (\omega )\omega }{\partial \omega }|}_{{\omega }_{0}}$ and the intensity of the transmitted pulse decays exponentially with the decay constant $2\kappa ({\omega }_{0}){\omega }_{0}={{N}_{\alpha }}^{-1}$ as a function of *N*. In our experiments, these values are ${{v}_{g}}^{-1}={\frac{\partial \eta (\omega )\omega }{\partial \omega }|}_{{\omega }_{0}}$ = 1.63 ns/stage and ${{N}_{\alpha }}^{-1}$ = 0.33/stage from the results shown in Fig. 4(f,h), in good agreement with the values of 1.73 and 0.41/stage, respectively, calculated using Eqs (1) and (5).

## Discussion

### Breakdown of traditional group velocity

The concept of traditional group velocity defined by Eq. (6) provides a good picture of the superluminal pulse velocity. However, this is limited to a small number of *N*. The traditional group velocity loses physical meaning as *N* becomes large. In the analyses of pulse propagation in a continuous Lorentz medium^28^, the propagation distances were classified in terms of the absorption length *α*. For the propagation distance, $z < {z}_{2}={\alpha }^{-1}{(\Delta {\nu }_{R}/\delta {\nu }_{p})}^{2}$, the traditional group velocity, ${v}_{g}={\rm{Re}}[\partial k/\partial \omega {|}_{{\omega }_{c}}^{-1}]$, properly describes the pulse propagation.

In our experiments, the number of resonator stages that could be studied was limited to six, due to excess losses in the recurrent system. We extended our study beyond this limit by carrying out numerical simulations of larger systems. The black line in Fig. 4(a) shows the transmission spectra as a function of the detuning frequency in the single-stage ring resonator used in the simulation. The two vertical arrows indicate the wavelengths at which pulse development was analyzed. The colored lines in Fig. 4(b) represent the calculated transmitted pulse profiles for different *N*, where the wavelength of the laser was adjusted to the resonance of the ring resonator (arrow 1). The pulse peak advanced because the ring resonator was prepared under under-coupling conditions $x < y$. In the region of *N* < 9, this advancement increased linearly, by 1.63 ns/stage; this suggests that the propagation distance was small enough for the traditional group velocity ${v}_{g}$ to be a suitable description in this case. In Fig. 4(f,h), the solid blue lines (line 1) represent the calculation of the temporal delay of the center of mass of the pulse and the normalized pulse intensity. On the other hand, in the region in which *N* > 9, the temporal profiles of the pulse became significantly deformed. The peak velocity, defined by the traditional definition of group velocity, loses its meaning. Thus, the expansion of Eq. (6) breaks down for large *N* and $z > {z}_{2}$. Figure 4(c) shows the transmitted spectrum calculated under the same conditions as used in Fig. 4(b). The central region of the spectrum was strongly attenuated, and the spectrum split into double peaks. The temporal structures in the *N* > 10 region shown in Fig. 4(b) can be interpreted as representing the interference between the double spectral peaks and the pulses, transformed into zero-area coherent optical pulses^16,37,38^.

### Superluminal to subluminal transition

#### Modified definitions of group velocity

In the traditional treatment, group velocity falls rapidly as the propagation distance increases. In continuous media, modified definitions of group velocity have been developed to model the pulse propagation in a Lorentz medium^28^. One such approach is the saddle-point method^31^, in which spectral shift during propagation is considered in terms of the drift of the saddle point during path integration in the complex plane. Peatross *et al*. proposed another idea, i.e., net and reshaping delay, in which the concept of conventional group velocity holds when applied to the surviving spectrum as opposed to the initial spectrum^32,33^. So far, discussion has been restricted to continuous and uniform media^28,31–33^. Here, we developed these ideas and examined how the pulse spectrum changes during pulse propagation through a serial array of resonators, and how this spectral shift affects the pulse propagation time.

Using the new definition of group velocity, a more interesting situation than resonance propagation [arrow 1 in Fig. 4(a)] arises when the incident laser frequency is detuned slightly from resonance, which corresponds to the anomalous dispersion region at the shoulder of the resonance line [arrow 2 in Fig. 4(a)]. The colored lines in Fig. 4(d) indicate the development of the transmitted pulse for different *N*. In the region of *N* < 15, the peak position moved towards the negative delay, indicating superluminal pulse propagation. Then, the pulse peak reversed and moved towards the positive delay with increasing *N*, indicating subluminal pulse propagation. Therefore, this behavior indicates that superluminal velocity only occurs over a small number of resonator stages, then transits to subluminal velocities after propagating beyond the critical number of resonators. In Fig. 4(f), the solid red line (line 2; overlapped with green circles) indicates the temporal delay of the center of mass as a function of *N*. The solid green line (line 3) shows the expected time delay for the case in which the pulse propagates with traditional group velocity, defined by Eq. (6).

In Fig. 4(g), the solid red line represents the results of the numerical simulation for the transmitted spectral peak, which moves towards the lower frequency region. When $\delta {\nu }_{p} \sim \Delta {\nu }_{R}$, the spectral components of the pulse show non-uniform absorption. For the spectral components in the central region of the resonance the absorption is strong and for the components in the wing region the absorption is weak. This asymmetric absorption results in a spectral shift of the transmitted spectrum. We denote the spectral profile and peak frequency after passing through *N* resonators as ${S}_{N}(\omega )$ and ${\omega }_{N}$, respectively. Although the spectral peak of the incident pulse ${\omega }_{0}$ lies in the anomalous region, when ${\omega }_{N}$ moves from the anomalous region to the normal region, the propagation velocity transits from a superluminal to a subluminal velocity. We can estimate the critical number of resonator stages for which the spectral shift cannot be neglected as ${N}_{c}=(\delta {\nu }_{p}/\Delta {\nu }_{R}){N}_{\alpha }$.

The solid green triangles in Fig. 4(f) show the expected time delays ${\tau }_{s}$ according to the saddle point method^28,31^, where the derivative of the group delay was taken at ${\omega }_{N}$ instead of the incident frequency ${\omega }_{0}$, i.e.,

8 $${{\tau }_{s}(N)=N\frac{\partial \eta (\omega )\omega }{\partial \omega }|}_{{\omega }_{N}}$$

The time delays calculated based on the saddle point method well describe the results, even where the traditional group velocity method ${\tau }_{g}$ fails completely, and shows the superluminal to subluminal transition in the propagation velocity. The open green circles in Fig. 4(f) show the expected time delays ${\tau }_{net}$ based on the net delay^32,33^:

9 $${\tau }_{net}(N)=\frac{{\int }_{-\infty }^{\infty }[\frac{\partial \eta (\omega )\omega }{\partial \omega }\cdot N]{S}_{N}(\omega )d\omega }{{\int }_{-\infty }^{\infty }{S}_{N}(\omega )d\omega }.$$

The net group delay is the spatial average of the conventional group delay weighted by the output spectral profile after passing through *N* resonators. We obtained good agreement by calculating the time delays based on the net delay, even in the case where *N* > 10. This is reasonable because the net delay employs the transmitted profile ${S}_{N}(\omega )$ directly in the calculation of the spatial average, while the saddle point method uses ${\omega }_{N}$ to represent the transmitted frequency.

## Summary

We have presented an experimental demonstration of the superluminal pulse propagation through a serial array of high-Q ring resonators. As the number of ring resonators increased, advancement of the pulse peak increased linearly, but the front edge of the pulse was neither advanced nor delayed in good accordance with the information velocity. We observed the superluminal to subliminal transition in our simulation. The treatment based on traditional group velocity loses physical meaning when the number of resonator stages increases beyond a certain value. The time delay calculated using the saddle point method and the net delay give a good representation, even when the traditional group delay fails completely. This demonstrates the superluminal to subluminal transition in the propagation velocity.

## Methods

**Figure 5:**
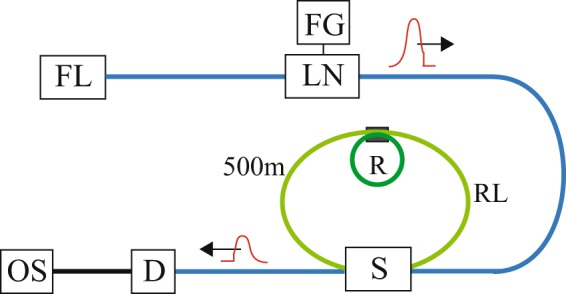
Schematic illustration of the experimental setup: *FL*, fiber laser; *LN*, LiNbO_3_ (LN) modulator; *FG*, function generator; *S*, 2 × 2 fast optical switch; *C*, coupler; *R*, ring resonator; *RL*, PM-fiber recurrent loop; *D*, InGaAs detector; *OS*, oscilloscope.

Our experimental setup is similar to the one described in ref.^16^. The setup is illustrated schematically in Fig. 5. An Er-fiber laser was used as the incident light source. The wavelength was 1,550 nm, and the laser frequency was fine-tuned by piezoelectric control of the cavity length of the fiber laser. The spectral width was 1 kHz and the average laser power was 0.1 mW. Smooth Gaussian-shaped pulses and pulses with sharp rising edges were produced using a LiNbO_3_ (LN) modulator. The bandwidth and extinction ratio of the LN modulator were 10 GHz and 10^−6^, respectively.

The dynamic recurrent system consisted of 2 × 2 fast optical switches and an ${L}_{RL}$ = 500 m polarization-maintaining (PM)-fiber as a recurrent loop. The rise and fall times of the optical switch were both 50 ns. When the incident optical pulse arrived at the optical switch, the switch opened over a time period of $T$ = 1,600 ns, and the optical pulse was injected into the recurrent loop. Then, the switch closed. We confined the pulse in the recurrent loop. The switch reopened at time ${T}_{N}=N\tau$, where $\tau ={L}_{RL}/c$ = 2,500 ns is the round trip time for the pulse in the recurrent loop and $N$ is the number of loop circulations. The pulse was then extracted from the recurrent loop into the transmission port after circulation. The transmission intensity through the system was observed using an InGaAs photodetector and reordered using a 600-MHz digital oscilloscope.

In our experiments, the number of resonator stages that could be studied was limited to six, due to excessive losses in the recurrent system. These losses arose from optical components additionally implemented in the experiment (coupler and isolator). Further, regarding the resonance condition, attenuation was strong due to the resonance absorption in the ring resonator. The implementation of gain in a recurrent loop may further increase the number of available resonator stages. The gain may, however, introduce a problem regarding the identity of the photons, as it could generate new photons that are not contained in the input pulses. Gain may also cause additional and unnecessary dispersion.

## Supplementary information

Supplementary Information
