# Supplementary material for: Development of superluminal pulse propagation in a serial array of high-Q ring resonators
Source: Sci Rep. 2019 Oct 3;9:14280. doi: 10.1038/s41598-019-50482-9 (PMC6776516; doi:10.1038/s41598-019-50482-9)
Supplement: Supplementary file 1 — Supplementary Information [file 41598_2019_50482_MOESM1_ESM.pdf]

Supplementary Information:

Development of superluminal pulse propagation in a serial array of high-Q ring resonators

Yuma Morita and Makoto Tomita*,
Department of Physics, Faculty of Science, Shizuoka University,
836, Ohya, Suruga-ku, Shizuoka, 422-8529, Japan

*Correspondence to: tomita.makoto@shizuoka.ac.jp

1 Pulse propagation under the off-resonance condition

In Fig. 2 in the main text, we experimentally examined superluminal pulse propagation in a serial array of ring resonators under the on-resonance condition. In this supplement, we examine pulse propagation under the off-resonance condition. Supplementary Fig. 1 shows experimental observations of the smooth Gaussian-shaped pulse transmitted through the serial array of ring resonators under different tuning conditions of the incident laser frequency. The ring resonator and the dynamic recurrent system were the same as those shown in Fig. 2 in the main text (under-coupling conditions, $x < y$). When the incident laser frequency was tuned within the anomalous dispersion region at the center of resonance, the advancement accumulated as the number of N increased (lines 1, 2), as discussed in the main text. On the other hand, when the laser frequency was tuned within the normal dispersion region at the wings of the resonance, the delay increased with the number of N (lines 4, 5). The transmitted pulse profiles N = 6 shown in Supplementary Fig. 1(e) demonstrate that the propagation times (delay and advancement) reflect the dispersion of the ring resonator, similar to the Lorentz absorption lines of an atomic gas.

The experimental results in Supplementary Fig. 1 thus show the analogues of pulse propagation between an atomic resonator and a serial array of resonators, emulating the propagation of pulses over different distances. The number of resonator stages that could be studied in our experiments was limited to six due to excess losses in the recurrent system. Therefore, the superluminal to subluminal transition was not observed in Supplementary Fig. 1, in good accordance with the simulation results shown in Fig. 4(a), with $N < 6$ (main text).

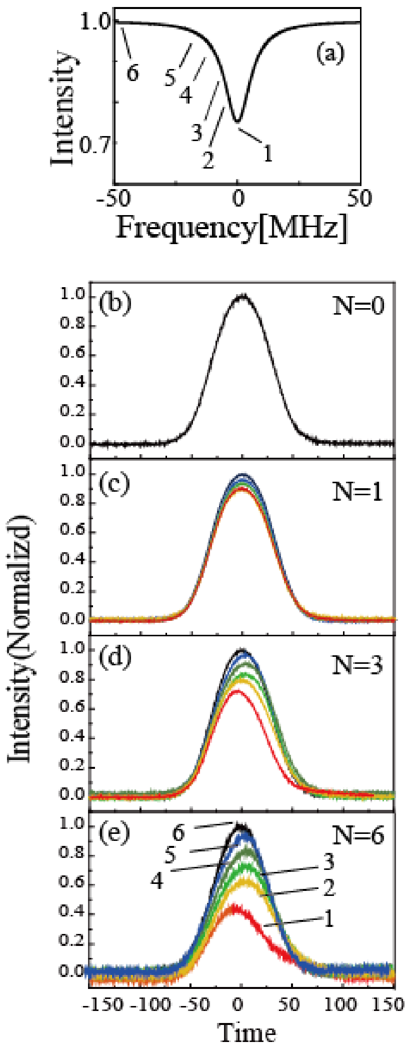

Supplementary Fig. 1. Experimental observations of the smooth Gaussian-shaped pulse transmitted through the serial array of ring resonators for different values of N . (a) Transmission spectra as a function of the detuning frequency in the single-stage ring resonator. Notations 1–6 indicate the frequencies at which the pulse propagation was analyzed. (b) $N = 0$ (input pulse), (c) 1, (d) 3, and (e) 6. The solid colored lines (lines 1–6) are the transmitted pulses observed at the incident frequency indicated in (a), respectively. The solid black line (line 6) corresponds to the input pulse (off-resonance). The solid red line (line 1) corresponds to the experimental data shown in Fig. 3 (on-resonance).

2 Front velocity in slow light

Many researchers have accepted that the true information is stored at the non-analytical points. The front edges of the pulses analyzed in the main text are one example of non-analytical points. We also investigated the edge propagation in other cases.

Supplementary Fig. 2 shows our experimental results. The left column shows the transmitted pulse profiles through the serial array of under-coupled (fast light) ring resonators, Supplementary Fig. 2 (a) shows the transmitted pulse profile of a smooth Gaussian-shaped input pulse and (b) Gaussian-shaped pulses on which the front edge was encoded on the leading side of the pulses, which correspond to the results shown in Figs. 2 (e1) and (e2), respectively, in the main text. Supplementary Fig. 2(c) shows the transmitted pulse profile, in which the edge was encoded in the trailing part of the pulse. In this case, the pulse peak was also advanced, but the sharp edge was neither advanced nor delayed.

The right column of Supplementary Fig. 2 shows the transmitted pulse profiles of the over-coupled resonator (slow light). We used a 80:20 coupler to achieve the over-coupling condition. In this case, the pulse peak was delayed, reflecting normal dispersion in the over coupled ring resonator. Supplementary Fig. 2 (e) shows the transmitted pulse profile of the Gaussian pulse, where the edge was encoded on the leading part of the pulse. A small spike appeared at $t_{NA} = -62$ ns, which was the position of the edge point. Supplementary Fig. 2 (f) shows the transmitted pulse when the edge was encoded on the trailing part of the pulse profile,

The advancement and delay in pulse peaks were accumulated depending on the coupling conditions of the serial array of ring resonators; however, the non-analytical points were neither advanced nor delayed. The non-analytical points can be interpreted as information; therefore, the experimental results agreed well with the idea that information velocity is equal to the velocity of light in a vacuum or the background medium, and is independent of the group velocity.

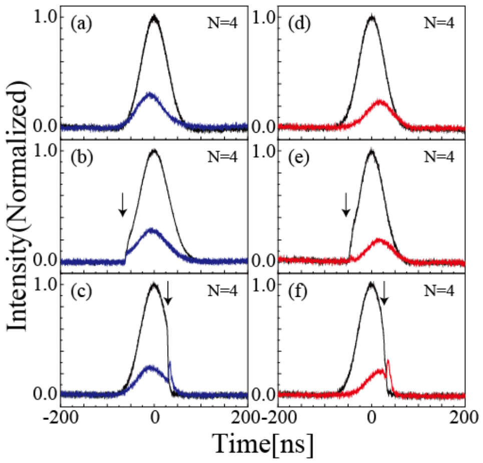

Supplementary Fig. 2. Temporal profiles of the transmitted pulse observed at N **= 4.** The solid black lines are input pulses (off-resonance). The colored lines are transmitted pulses (on-resonance). The left and right columns show the observations obtained using under- (fast light) and over-coupled (slow light) ring resonators, respectively. The input pulses were as follows: (a) and (d) show results obtained with smooth Gaussian-shaped pulses; (b) and (e) show results obtained with Gaussian-shaped pulses in which the front edge was encoded on the leading side of the pulses; and (c) and (f) show results obtained with Gaussian-shaped pulses in which the end edges were encoded on the trailing edges of the pulses. The downwards pointing arrows indicate the positions of the edges.
